# Frozen Section Diagnostic Pitfalls of Sertoli-Leydig Cell Tumor with Heterologous Elements

**DOI:** 10.1155/2018/5151082

**Published:** 2018-07-25

**Authors:** Ashley Burris, Caleb Hixson, Nathaniel Smith

**Affiliations:** ^1^Brooke Army Medical Center, San Antonio, TX, USA; ^2^Tripler Army Medical Center, Honolulu, HI, USA

## Abstract

A woman in her sixth decade presented with several months of abdominal cramping, decreased appetite, bloating, and increased constipation. Radiologic imaging revealed a 28 cm, multilocular, heterogeneous cystic neoplasm involving the right adnexa. An intraoperative frozen section showed mucinous glandular epithelium, with and without foci of goblet cells, embedded in apparent ovarian stroma. The findings were concerning at least borderline mucinous cystadenoma with possible invasion. Subsequent surgical management and staging were performed. Permanent sections showed a moderately to poorly differentiated Sertoli-Leydig cell tumor (SLCT) with retiform foci and heterologous elements. The discrepancy between frozen and permanent sections was attributable to solely sampling a focus of heterologous elements during intraoperative consultation. The rarity of SLCT and even rarer presence of both heterologous and retiform elements make this concerning frozen section diagnostic pitfall.

## 1. Background

Sertoli-Leydig cell tumors (SLCT) are rare ovarian neoplasms which may present a diagnostic challenge at the time of intraoperative consultation with pathology. In addition to a wide array of histological patterns, these tumors may also contain heterologous elements including mucinous glands, skeletal muscle, and chondroid differentiation. Consequently, solely sampling heterologous elements may lead to a confounding diagnostic differential. Here, we report a case of a Sertoli-Leydig cell tumor with both heterologous and retiform elements in which only heterologous components were sampled during intraoperative consultation. We also review Sertoli-Leydig cell tumor characteristics and methods to avoid this potential diagnostic pitfall.

## 2. Case Presentation

A woman in her sixth decade presented with voice alterations, abdominal cramping, decreased appetite, bloating, and increased constipation for several months. A family history included a maternal aunt and grandmother with ovarian cancer. Physical exam revealed virilization and clitoromegaly but it was otherwise noncontributory.

## 3. Investigations

An endovaginal ultrasound showed a large complex right adnexal mass with internal vascular flow measuring 20 cm in greatest dimension. A subsequent CT with contrast showed a large, multilocular, heterogeneous cystic structure involving the right adnexa and extending into the upper abdomen to the level of the liver. Serological studies showed cancer antigen 125 (CA-125) was elevated at 80.5 U/mL (reference range 0.6-35.0 U/mL). Inhibin and other sex hormone serologies were not performed preoperatively.

**Figure 1:**
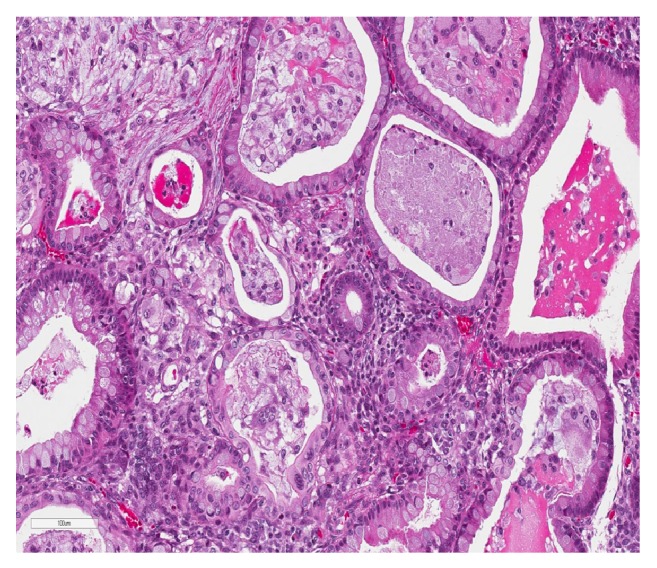
Heterologous elements on frozen section showing solely mucinous epithelium with angulated glands concerning invasion but without a definitive desmoplastic response; H&E at 200x.

Gross dissection at the time of intraoperative consultation revealed a 28 cm, 2300-gram red-pink multiloculated cystic neoplasm containing seromucinous fluid and focal papillary excrescences. A representative frozen section from an area of papillary architecture showed small- to medium-sized intestinal-type mucinous epithelium in a background of mixed inflammation and necrotic debris. A few of the glands were angulated and concerning invasion but lacked a definitive desmoplastic response (Figure 1). A diagnosis of “multicystic seromucinous neoplasm, at least borderline, suspicious for invasion” was rendered. Surgical management and staging included a bilateral salpingo-oophorectomy, appendectomy, regional lymph node dissection, and omental and peritoneal biopsies.

**Figure 2:**
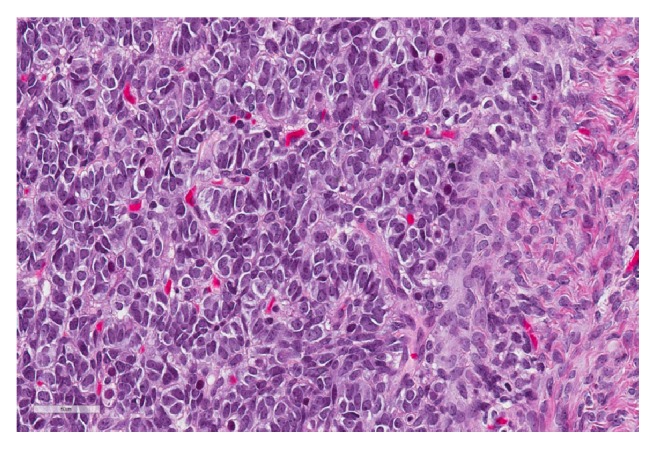
Areas of trabecular growth composed of Sertoli cells with ovoid nuclei, punctate nucleoli, and nuclear molding; H&E at 400x.

**Figure 3:**
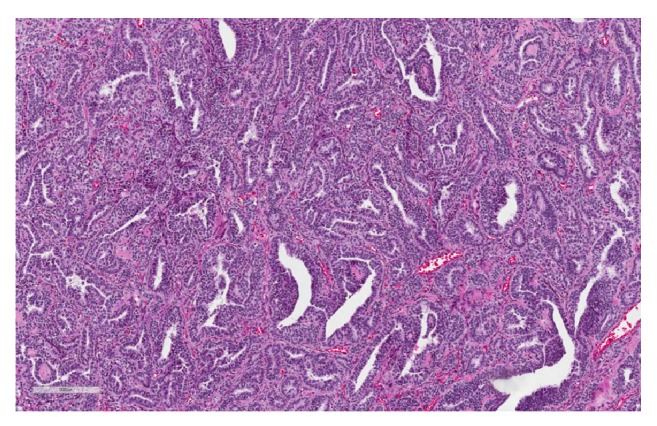
Retiform pattern with “staghorn” slit-like tubules; H&E at 100x.

**Figure 4:**
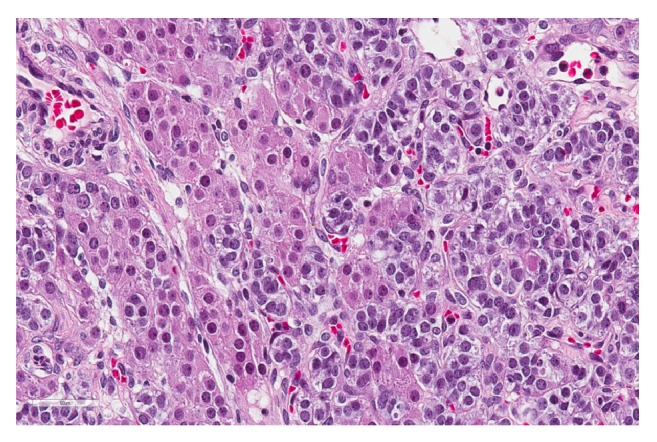
Nests of Leydig cells with abundant eosinophilic cytoplasm and round nuclei; H&E at 400x.

Permanent sections of the tumor showed a wide array of morphological patterns. The cells were arranged in multiple patterns including trabeculae, tubules, sheets, and papillary structures lining cystic areas. A minor component of the neoplasm was comprised of heterologous elements including mature bland mucinous glands resembling both endocervical and intestinal epithelium in association with vague chondroid-like areas. The predominant cell population was characterized by ovoid nuclei lacking appreciable grooves, lightly basophilic cytoplasm, and punctate nucleoli consistent with Sertoli cells (Figure 2). A distinct retiform pattern was also identified consisting of irregular networks of elongated and slit-like tubules with a “staghorn” appearance (Figure 3). A second population of Leydig cells with round nuclei and abundant eosinophilic cytoplasm was interspersed throughout the neoplasm (Figure 4).

## 4. Differential Diagnosis

The differential diagnosis at frozen section is quite broad owing to the wide variety of histological patterns of SLCT. An endometrioid carcinoma with sertoliform features may mimic SLCT given the overlapping histological features of expanded tubules, clustered glands, and primitive appearing squamous cells in a possible background of luteinized stroma. Nonetheless, SLCT tends to present as a unilateral tumor in a younger age group with clinically detectable virilization [1, 2].

Clinically, yolk sac tumors (YST) can mimic SLCT as both often present as large unilateral adnexal neoplasms in younger females and may show elevations in alpha fetoprotein (AFP) [3]. The AFP serum elevation is often higher in YST and is a characteristic also exhibited immunohistochemically. Despite papillary arrangements and a myriad of histologic variants in both tumors, AFP is typically strongly and diffusely positive in YST in contrast to only focal positivity confined to the hepatoid component of SLCT [3].

Granulosa cell tumors are less likely to enter the clinical differential given the manifestations of increased estrogen production as opposed to the virilizing androgens seen in SLCT. However, both neoplasms may show architectural overlap including sheets and trabeculae of cells exhibiting inhibin positivity [1]. Histologic characteristics such as nuclear grooves and Call-Exner bodies of granulosa cell tumor are important clues in the differential diagnosis. Additionally, heterologous elements strongly argue against a diagnosis of granulosa cell tumor [4].

The most confounding differentials arise if only heterologous elements are sampled during frozen section. As in this case, mucinous intestinal epithelium may mimic a primary mucinous tumor of the ovary and a metastatic mucinous neoplasm from organs such as the appendix. Other mature mesenchymal components such as cartilage and skeletal muscle may also be easily mistaken for components of a mature teratoma.

## 5. Outcome and Followup

The final diagnosis was “Sertoli-Leydig Tumor, moderately to poorly-differentiated with retiform foci and heterologous elements”. The contralateral ovary, appendix, lymph nodes, and omental/peritoneal biopsies were negative for involvement. Chemotherapeutic treatment with Bleomycin was initiated and followup to date has shown no local or metastatic tumor recurrence.

## 6. Discussion

To our knowledge, this is the first reported case of an intraoperative consultation on a Sertoli-Leydig cell tumor in which only heterologous elements were sampled at the time of frozen section. Sertoli-Leydig cell tumors (SLCT) are a rare subtype of sex-cord stromal tumors with an incidence of less than 0.5% of ovarian neoplasms [1]. Although the average age at presentation is skewed by outliers, the tumor typically presents in the third decade of life [2, 5–7]. The retiform variant presents in younger patients and typically presents within the second decade [7].

Clinical manifestations of SLCT result from excessive tumor growth and endocrine hormone production. Tumor growth has been reported to exceed 30 cm with accompanying weights well over 2000 grams. The tumor burden results in a palpable mass on physical examination and secondary nonspecific pains [2, 5, 8]. Potential clinical signs also include menstrual irregularities, dysmenorrhea, and virilization resulting from androgen dysregulation [2, 5, 6, 9].

A thorough review of the patient's radiographic studies prior to the intraoperative consult may aid in macroscopic examination. The preferred initial imaging technique is transvaginal color Doppler sonography which usually reveals a solid and cystic neoplasm with increased neovascular blood flow [10]. The gross features of SLCT are variable and range from smooth-walled cysts to papillary, spongy excrescences and firm nodules. The presence of mucinous heterologous elements may affect the gross appearance and consistency of the cystic fluid. Prominent mucinous heterologous elements will likely increase the viscosity of the fluid while cartilage or bone may be misinterpreted as a component of a teratoma [2, 3, 5, 8].

Microscopically, an SLCT will have varying proportions of both Sertoli and Leydig cells. A “sertoliform” pattern is characterized by tubules, cords, and trabeculae of cells with round to oval nuclei and indistinct nuclei whereas Leydig cells are polygonal cells with abundant eosinophilic cytoplasm and centrally placed nuclei occurring in sheets or nests (Figures 2 and 4).

The retiform variant of SLCT histologically resembles the rete testes and is comprised of dilated and tortuous tubules with a “staghorn-like” appearance (Figure 3). This variant may also contain cystic areas with edematous papillae and micropapillae resembling serous borderline tumor. The retiform variant is exceptional among sex-cord tumors because it has no definitive testicular counterpart and likely arises from a more primitive cell type [7, 8, 11]. As such, retiform morphology is seen more commonly in intermediate to poorly differentiated SLCT. Heterologous elements may also be seen and are typically composed of endodermal and mesodermal components such as gastrointestinal epithelium, skeletal muscle, and areas of chondroid differentiation.

## Additional Points

*Learning Points*. (i) Sertoli-Leydig cell tumors (SLCT) are a rare subtype of sex-cord stromal tumors which typically present with signs of virilization in females in the second or third decade. (ii) SLCT presents with a wide array of variable histological features. The possibility of sampling only heterologous elements may lead to an erroneous differential. (iii) A thorough review of the patient's clinical manifestations and open communication between pathologist and surgeon may provide the best initial assessment of the diagnosis even before microscopic evaluation. (iv) Although rare, pathologists should be aware of these potential diagnostic pitfalls when examining ovarian tumors which show mucinous, cartilaginous, and/or papillary features.
